# Hopelessness and alcohol use: The mediating role of drinking motives and outcome expectancies

**DOI:** 10.1016/j.abrep.2016.11.001

**Published:** 2016-11-05

**Authors:** Laura Baines, Andrew Jones, Paul Christiansen

**Affiliations:** Department of Psychological Sciences, University of Liverpool, United Kingdom; UK Centre for Tobacco and Alcohol Studies, United Kingdom

**Keywords:** Alcohol, Outcome expectancies, Anxiety-sensitivity, Coping, Hopelessness

## Abstract

**Introduction:**

Heavy drinking is a considerable public health concern. There is a broad evidence-base examining the separate contributions of personality characteristics, motives and alcohol-expectancies on subsequent alcohol use to identify those at risk. However, little is known about the complex relationships by which these variables may interact to predict drinking behavior. Feelings of hopelessness and anxiety sensitivity are hypothesized to be distal predictors of alcohol use, with outcome expectancies and drinking motives more proximal. Therefore, the aim of the current study was to examine whether hopelessness and anxiety sensitivity influenced alcohol use via drinking to cope and alcohol - outcome expectancies.

**Methods:**

We recruited 230 participants to complete an online questionnaire consisting of the brief drinking motives questionnaire, the Substance Use Risk Profile scale and Brief Comprehensive Effects of Alcohol scale. We conducted path analyses using structural equation modelling.

**Results:**

We demonstrated a significant direct effect of anxiety sensitivity on alcohol use, and a significant serial indirect effect of hopelessness through coping motives and alcohol outcome expectancies.

**Conclusions:**

These findings suggest feelings of hopelessness may predict alcohol consumption through a complex pathway and future research should use these findings to identify individuals at risk of increased alcohol use.

## Highlights

• There was a direct relationship between Anxiety sensitivity and alcohol use.
• There was a complex relationship between hopelessness and alcohol use.
• Findings help to explain inconsistencies in the literature.

## 1 Introduction

Heavy drinking constitutes a significant public health concern, directly costing the UK National Health Service approximately £3.5bn per year (Public Health England, 2014). A considerable amount of research has demonstrated that certain personality traits are associated with elevated alcohol use, for example, impulsivity (a tendency to act without thinking; (Dawe, Gullo, & Loxton, 2004)) and neuroticism (a tendency to feel psychological distress including anxiety and depression; (Costa & McCrae, 1992). As well as these non-substance-specific traits, measures of specific substance-related dispositions have been developed to improve our understanding of the individual differences that may contribute to alcohol use.

The Substance Use Risk Profile Scale (SURPS; Woicik, Stewart, Pihl, & Conrod, 2009) was developed to examine four motivational profiles for alcohol use, measuring Anxiety Sensitivity, Hopelessness, Sensation Seeking and Impulsivity. Hopelessness - pessimism towards oneself and one's future, often co-occurring with depression (Hudson, Wekerle, & Stewart, 2015) and anxiety sensitivity - awareness of symptoms which causes distress (Loxton, Bunker, Dingle, & Wong, 2015), both have been recognized in the four-factor model of personality vulnerability to alcohol misuse (Castellanos-Ryan & Conrod, 2012). This model predicts that each personality risk factor is related to specific drinking motives (Mackinnon, Kehayes, Clark, Sherry, & Stewart, 2014) and precise patterns of substance use, as well as certain psychopathological disorders (Castellanos-Ryan & Conrod, 2012).

Support for the four-factor model has been found in several studies demonstrating that these personality risk factors predict unique variance in alcohol consumption (Hustad, Pearson, Neighbors & Borsari, 2014). However, the overall evidence is equivocal. Research has found positive associations between anxiety-sensitivity and alcohol use (e.g. Omiya, Kobori, Tomoto, Igarashi, & Iyo, 2015) or problems (e.g. (Mackinnon et al., 2014), but negative associations have also been reported (Ali et al., 2016, Castellanos-Ryan et al., 2013, Krank et al., 2011, Wagner, 2001). Additionally, hopelessness has been found to positively correlate with alcohol use in several studies (e.g. Krank et al., 2011, Malmberg et al., 2010), whereas no association was reported by Mackinnon et al. (2014). More recently, (Loxton et al., 2015) failed to find an association between both hopelessness or anxiety sensitivity and drinking behaviour. Currently, the strength and direction of these relationships are unclear (Staiger, Kambouropoulos, & Dawe, 2007), and this is most likely to be due to individual differences in variables that mediate the association between these personality types and alcohol mis(use).

Motivational models of alcohol use argue that risk factors, including personality traits, may influence alcohol use through a common pathway of drinking motives (Stewart & Devine, 2000). These motives include; social, enhancement, conformity and coping drinking motives (Cooper, 1994). Although all these motives are consistently found in samples of drinkers, and are to some extent associated with alcohol use, drinking to cope (drinking to reduce or evade anxiety and negative affect; (Blumenthal, Leen-Feldner, Frala, Badour, & Ham, 2010)) and drinking for enhancement (drinking to enhance or sustain positive feeling; (Lewis et al., 2008)) are more frequently associated with heavy alcohol use (Tobin, Loxton, & Neighbors, 2014). Indeed, coping motives are associated with a greater number of drinking problems (Thomas, Merrill, von Hofe, & Magid, 2014) and other alcohol related consequences, such as risky behaviour and academic/occupational problems (Merrill & Read, 2010). Importantly, the four-factor model of personality vulnerability to alcohol misuse argues that individuals high in hopelessness or anxiety sensitivity may drink to cope (Schlauch et al., 2014) as anxiety-sensitivity increases drinking due to its perceived stress relieving effects, whereas hopelessness increases drinking to cope with negative affect (Castellanos-Ryan & Conrod, 2012).

The indirect effect of anxiety sensitivity and/or hopelessness on drinking through coping motives has been demonstrated in numerous studies (e.g. Grant et al., 2007, Mackinnon et al., 2014, Schlauch et al., 2014, Stewart et al., 2001, Woicik et al., 2009)). There are, however, multiple examples of studies that fail to show one or both of these indirect effects (e.g. (Mackinnon et al., 2014, Magid et al., 2007, Merrill and Read, 2010). This inconsistency suggests that there are additional factors mediating the association between anxiety sensitivity/hopelessness and alcohol misuse. One factor that has also been implicated as a mediator between personality and drinking is alcohol outcome expectancies (AOE; (Donovan, Molina, & Kelly, 2009). These refer to what drinkers believe or expect will happen when they consume alcohol. Specifically, positive AOE are beliefs that drinking alcohol may be beneficial and lead to positive outcomes for the drinker (Blume & Guttu, 2015). Much research has shown AOE, particularly positive, are associated with alcohol use (Blume and Guttu, 2015, Cable and Sacker, 2008, McCarthy et al., 2000, Patrick et al., 2010, Reich et al., 2012) as well as coping motives (Carrigan, Ham, Thomas, & Randall, 2008). Importantly, studies have shown that coping strategies and AOE interact to predict alcohol use (Hasking & Oei, 2002), and that both AOE and coping motives may be required to significantly predict drinking (e.g. (Cooper, Russell, & George, 1988)). Therefore, it is possible that both coping motives and AOE are mediators in the relationship between hopelessness, anxiety-sensitivity and alcohol use.

The aim of this study was to examine the potential pathway by which hopelessness and anxiety sensitivity contribute to alcohol use in social drinkers. We hypothesized that hopelessness and anxiety-sensitivity would be associated with drinking to cope. We also hypothesized that coping motives and positive AOE would be associated, and both of these were expected to predict increased alcohol use. Finally, we hypothesized that both coping drinking motives and positive AOE would mediate the indirect effect of hopelessness and anxiety sensitivity on alcohol use.

## 2 Methods

### 2.1 Participants

Two-hundred and thirty participants (196 female), with a mean age of 22.91 (± 9.68) years, were recruited from the university and wider community. Inclusion criteria involved a minimum age of 18 years, regular consumption of alcohol (at least once per week) and fluent English speaking. Data was collected using opportunity sampling. Participants were recruited via university intranet, social media and advertisements in the community. All participants provided informed consent before completing the survey, which was approved by the University of Liverpool's Research Ethics Committee.

### 2.2 Questionnaires

*Time Line Follow-Back* (TLFB; Sobell & Sobell, 1990): The TLFB self-report questionnaire was used to assess weekly alcohol consumption. Following an explanation of the number of units contained in standard alcoholic drinks (one UK alcohol unit = 8 g of alcohol), participants estimated the number of units they had consumed over the preceding seven days. Although this represents a short period of time, these periods can be used to assess unit consumption with minimal loss in accuracy of data (Gioia et al., 2012, Vakili et al., 2008).

*The Alcohol Use Disorders Identification Test* (AUDIT; (Saunders, Aasland, Babor, De la Fuente, & Grant, 1993)): The AUDIT was used to assess hazardous drinking. The AUDIT consists of ten fixed response questions regarding alcohol consumption and consequences of drinking. Scores on the AUDIT range between 0 and 40 with scores of 8 or above indicating hazardous or harmful alcohol use.

*The Substance Use Risk Profile Scale* (Woicik et al., 2009). The SURPS has 23 items measuring four personality risk factors (sensation seeking, impulsivity, hopelessness and anxiety-sensitivity) for alcohol misuse. Sensation seeking is measured on six items, impulsivity on five, hopelessness on seven and anxiety sensitivity on five. Answers took the form of a four-point Likert scale ranging from strongly disagree to strongly agree.

#### 2.2.1 *Brief comprehensive effects of alcohol scale* (CEOA-B; Ham, Stewart, Norton, & Hope, 2005)

This consisted of 15 items measuring what participants expect to happen when they consume alcohol (i.e. alcohol outcome expectancies). The scale contains positive AOE subscales (Tension reduction; Social facilitation; Liquid courage; Self Perception) and negative expectancy subscales (Cognitive-behavioural impairment; Risk taking/aggression; negative self evaluation). All statements were a possible completion of the sentence “when I drink alcohol, I expect that…” Answers took the form of a four-point scale from strongly disagree to strongly agree.

#### 2.2.2 *Modified drinking motives questionnaire short form* (DMQ-R SF; Kuntsche & Kuntsche, 2009)

The DMQ-R SF is a 12 item self-report scale in which participants endorse statements relating to different motivations to drink on a Likert scale. Answers range from 1 (never/almost never) to 5 (always/almost always). The DMQ-R consists of 4 subscales; Conformity, Enhancement, Social and Coping.

### 2.3 Procedure

After accessing the online site, participants were shown an information sheet and gave informed consent. Participants were then asked to complete the questionnaires and give basic demographic information (age and gender). Participants were debriefed and thanked for participation.

### 2.4 Data analysis

We computed a composite measure of alcohol use as our dependent variable. We used this measure in order to better capture the general pattern of alcohol use rather than a specific behaviour such as heavy episodic drinking, as in previous research (see (Christiansen and Bloor, 2014, Fernie et al., 2013)). This consisted of scores on the AUDIT, units consumed as measured by the TLFB and frequency of heavy episodic drinking (6 + units in a single session for females 8 + for males: Office of National statistics 2015), z-scored and combined. A Principal Component Analyses confirmed that these three separate measures of alcohol involvement loaded onto a single component (eigenvalue = 2.28; accounting for 76.07% of variance) with all factor loadings ≥ 0.79. Before analysis of the structural model, all questionnaire variables were square root transformed. Multiple indices of model fit were calculated to ensure that the model represented a good fit of the data. Normed χ^2^ values were calculated (χ^2^/*df*). χ^2^/*df* values between 1 and 5 are indicative of an acceptable model fit (Schumacker & Lomax, 2004). The SRMR absolute fit index was also used to assess model fit, as this measure is less affected by sample size distribution and kurtosis as it is not a simple variation of χ^2^, SRMR values under 0.08 are representative of a good model fit. As well as using the aforementioned discrepancy function methods, model fit was also estimated using non-centrality based indices. Specifically, the comparative fit index (CFI) and root mean square error of approximation (RMSEA). CFI values equal to > 0.95 and RMSEA values equal to or lower than 0.06 were used a cut offs for good fit (Hu & Bentler, 1999). Adequate fit for CFI and RMSEA values are > 0.90 and lower than 0.08 respectively (Browne & Cudeck, 1989). In describing specific relationships within the model we report standardised regression coefficients in the figure. In addition, bias corrected bootstrapping was utilised to assess overall indirect effects of personality on alcohol use. Finally, in order to obtain specific indirect effects for the hypothesised serial mediation (personality-coping-positive-AOEs to alcohol misuse) we utilised PROCESS (Hayes, 2012), to allow the computation of a regression coefficient and asymmetrical bootstrap confidence intervals for the indirect effect.

## 3 Results

For descriptive statistics of the sample see Table 1.

**Table 1:** Descriptive statistics.

Measure	Mean (± SD)
AUDIT	11.93 (± 5.82)
UK weekly units	16.87 (± 14.43)
Binge frequency	1.91 (± 0.73)
Coping motives	6.04 (± 2.68)
CEOA sexual enhancement	2.34 (± 0.85)
CEOA tension reduction	2.19 (± 0.71)
CEOA social facilitation	3.55 (± 0.58)
CEOA liquid courage	3.07 (± 0.70)

Legend: AUDIT = Alcohol Use Disorders Identification Task; CEOA = Comprehensive Effects of Alcohol.

### 3.1 Structural model (Fig. 1)

**Fig. 1:**
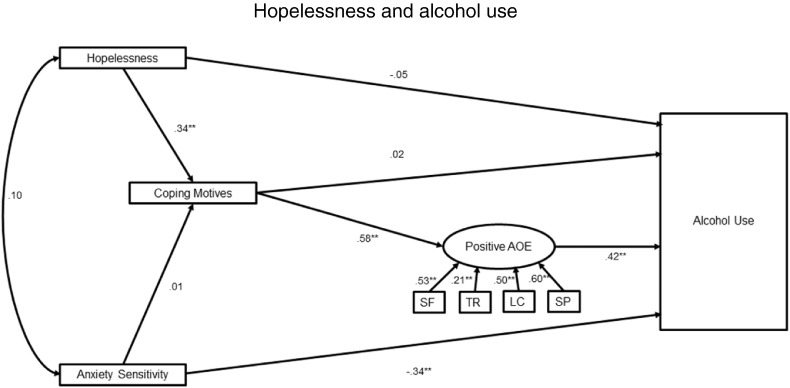
The indirect effects of hopelessness and anxiety sensitivity on alcohol use via coping motives and positive AOEs (standardised regression coefficients presented) ^⁎^*p* < 0.001: LC = Liquid courage; SF = Social facilitation; SP = Self-perception; TR = Tension reduction.

CFA of the positive AOE variable was found to be a good to acceptable fit on all measures (χ^2^/*df* = 2.06; SRMR = 0.07; CFI = 0.90; RMSEA = 0.07). The structural model was found to be good fit for the data (χ^2^/*df* = 1.42; SRMR = 0.05; CFI = 0.96; RMSEA = 0.04). As can be seen from Fig. 1 there was a significant direct effect of anxiety sensitivity on alcohol misuse (with increased anxiety sensitivity being associated with reduced alcohol use *p* < 0.001) although anxiety sensitivity had no indirect effect on alcohol use (95% CI = − 0.07 to 0.10, *p* = 0.84). There was no direct effect of hopelessness on alcohol misuse, although hopelessness was associated with coping motives (*p* < 0.001). Similarly coping motives did not directly predict alcohol misuse but did predict positive AOEs (*p* < 0.001), which, in turn, predicted increase alcohol use (*p* = 0.001). Overall, there was a significant indirect effect of hopelessness on alcohol use (95% CI = 0.08 to 0.26, *p* = 0.01).

Further analysis of the indirect effect of hopelessness on alcohol use using PROCESS revealed that there was a significant in serial direct effect through coping motives and alcohol outcome expectancies (B = 0.23, SE = 0.08; 95% CI = 0.11 to 0.43). The simple indirect effect of hopelessness via coping (B = 0.23, SE = 0.15; 95% CI = − 0.05 to 0.54), or by positive AOEs (B = − 0.07, SE = 0.11; 95% CI = − 0.32 to 0.10), were not significant. Importantly, the serial indirect effect was maintained when anxiety sensitivity, negative AOEs and gender were controlled for in the model (B = 0.16, SE = 0.06; 95% CI = 0.07 to 0.31).

## 4 Discussion

The current study investigated the extent to which the relationship between anxiety sensitivity, hopelessness and alcohol use is mediated by drinking motives and positive AOE. Structural equation modelling revealed that anxiety-sensitivity, but not hopelessness, had a direct effect on alcohol use. Hopelessness did however, have a significant indirect effect on alcohol use via coping and positive AOE; critically the simple indirect effects of hopelessness on alcohol use via coping or positive AOE were non-significant. Hopelessness was related to coping motives and although coping motives did not directly predict alcohol misuse, they did predict positive AOE, which in turn predicted increased alcohol use.

The direct negative association between anxiety-sensitivity and alcohol use has been previously reported (Ali et al., 2016, Castellanos-Ryan et al., 2013, Krank et al., 2011, Wagner, 2001). Unexpectedly, we did not find any relationships between anxiety-sensitivity and coping motives, thus no indirect effects were found. This is supported by previous research (Loxton et al., 2015, Mackinnon et al., 2014), which also found no indirect effect through coping motives and suggests that individuals who experience high anxiety-sensitivity may be avoidant of alcohol use. Taken together, these findings along with several others (Mackinnon et al., 2014, Omiya et al., 2015, Stewart and Kushner, 2001) do not support the four-factor model of alcohol use. Importantly, it has been suggested that the effect of anxiety sensitivity on drinking may be more complicated compared to other personality factors. Psychological, physical and social concerns are said to be three lower-order components of anxiety sensitivity, all of which may have unique associations with various aspects of substance use (Stewart & Kushner, 2001) Thus, this could be beneficial to recognise when developing theoretical models and potential future research.

We demonstrated no direct effect of hopelessness on alcohol use, (see also Loxton et al., 2015). Nevertheless, hopelessness was associated with increased coping motives, as predicted by the four-factor model, and also replicates several studies (e.g. (Mackinnon et al., 2014, Woicik et al., 2009)). Furthermore, although coping motives did not directly predict alcohol misuse, they did predict positive AOE, replicating previous findings (Carrigan et al., 2008). In turn, positive AOEs predicted increased alcohol use (see also, Blume and Guttu, 2015, McCarthy et al., 2000). Taken together these results demonstrate a complex, indirect effect of hopelessness on alcohol use through coping motives and then positive AOE. This suggests that a high risk of alcohol misuse in individuals high in hopelessness may only be present if they are drinking to cope (Hudson et al., 2015) and, critically, expect drinking alcohol will have a positive outcome. These findings highlight the critical role AOEs play in predicting alcohol use and are consistent with other research that argues both drinking motives and AOE are mediators between personality risk factors and alcohol use (e.g. Urbán, Kökönyei, & Demetrovics, 2008). Moreover, the simple indirect effects via coping motives or positive AOE were not significant. Although contrary to some studies (Mackinnon et al., 2014, Stewart et al., 2001), these findings support literature (Cooper et al., 1988, Hasking and Oei, 2002) that demonstrate coping strategies and AOE interact or are both required to significantly predict alcohol use.

The results of this study should be interpreted in light of the limitations. Our composite measure of alcohol use did not allow for us to make inferences on specific drinking behaviours (e.g. heavy episodic drinking). The study also used a cross-sectional design, which does not allow for us to track the dynamic relationship between these variables over time. Our sample was also older than typical UK university cohorts (generally 18–21 years), due to our recruitment from the wider community. Whilst this increases the generalizability of our findings we are unable to examine differences in student vs community drinking. In relation to this, our recruitment led to an under-representation of males in our sample. Therefore, future research should aim to recruit more representative samples and examine differences in student vs non-students, as well as changes in the relation between these variables over time. Should this pathway be consistent and robust, it may contribute to the development of personality-targeted prevention programs, which have been shown as successful in reducing alcohol use (Newton et al., 2016). Future research should also investigate the relationship between the three components of anxiety-sensitivity and alcohol use. This may improve understanding of the complex relationship between anxiety-sensitivity and drinking and also go some way to explain the lack of coherence in the literature.

## 5 Conclusions

To conclude, the current study demonstrates the importance of coping motives and AOEs when exploring the association between hopelessness and alcohol use. Critically we found that individuals high in hopelessness, who are drinking to cope and also hold positive AOEs, should be recognised as high risk for hazardous alcohol use. This suggests that it may be beneficial for targeted interventions to emphasise other ways of coping with anxiety or depression that are incompatible with drinking, and to increasingly focus on changing positive AOE that people high in hopelessness hold with relation to alcohol.

## Role of funding sources

This research did not receive any specific grant from funding agencies in the public, commercial, or not-for-profit sectors.

## Contributors

LB and PC developed the study. LB collected the data. PC analysed the data. LB and AJ drafted the manuscript. All authors contributed to the final version.

## Conflict of interest

The authors have no conflicts of interest.
